# Aftermath of bustamante attack on genomic beacon service

**DOI:** 10.1186/s12920-017-0278-x

**Published:** 2017-07-26

**Authors:** Md Momin Al Aziz, Reza Ghasemi, Md Waliullah, Noman Mohammed

**Affiliations:** 1 Department of Computer Science, University of Manitoba, Winnipeg, Canada; 2 Department of Mathematics, Faculty of Sciences, Bu-Ali Sina University, Hamedan, Iran

**Keywords:** Bustamante attack, Genomic beacon service, GA4GH genomic beacon, Bustamante attack mitigation, Human genomic data privacy, Re-identification attack

## Abstract

**Background:**

With the enormous need for federated eco-system for holding global genomic and clinical data, Global Alliance for Genomic and Health (GA4GH) has created an international website called beacon service which allows a researcher to find out whether a specific dataset can be utilized to his or her research beforehand. This simple webservice is quite useful as it allows queries like whether a certain position of a target chromosome has a specific nucleotide. However, the increased integration of individuals genomic data into clinical practice and research raised serious privacy concern. Though the answer of such queries are yes or no in Bacon network, it results in serious privacy implication as demonstrated in a recent work from Shringarpure and Bustamante. In their attack model, the authors demonstrated that with a limited number of queries, presence of an individual in any dataset can be determined.

**Methods:**

We propose two lightweight algorithms (based on randomized response) which captures the efficacy while preserving the privacy of the participants in a genomic beacon service. We also elaborate the strength and weakness of the attack by explaining some of their statistical and mathematical models using real world genomic database. We extend their experimental simulations for different adversarial assumptions and parameters.

**Results:**

We experimentally evaluated the solutions on the original attack model with different parameters for better understanding of the privacy and utility tradeoffs provided by these two methods. Also, the statistical analysis further elaborates the different aspects of the prior attack which leads to a better risk management for the participants in a beacon service.

**Conclusions:**

The differentially private and lightweight solutions discussed here will make the attack much difficult to succeed while maintaining the fundamental motivation of beacon database network.

**Electronic supplementary material:**

The online version of this article (doi:10.1186/s12920-017-0278-x) contains supplementary material, which is available to authorized users.

## Background

Recent improvements on Genomic data sharing efforts have led researchers and clinicians gaining access and make comparisons across data from millions of individuals. Such development made it easier for genetic variant interpretation and in some cases treatment of rare diseases such as some special cancer types [1]. Most of the big organisations i.e., Broad institute in the U.S., BGI in china, Wellcome Trust Sanger in the UK etc. have an interest of making DNA data easier to access in order for their researchers to treat patients one on one. However, after twelve years of completion Human Genome project, the tremendous growth of genomic data has exceeded the containers build to hold such data. Genomic and clinical data are generally still collected in either by disease, institution or by country. More importantly, current data sharing privacy requirements do not necessarily protect individuals identity within and across institutions and countries. Furthermore, data often stored in incompatible file format and there are no standardized tools and analytical methods are in place [1–3].

With such tremendous needs for global genomic and clinical data repository system, Global Alliance for Genomic and Health (GA4GH) has created a federated data ecosystems called Beacon data network, a way for searching genomic data as simple as World Wide Web. Since the project’s launch in the middle of 2015, the beacon network has currently 23 different organizations covering over 250 genomic datasets. The data sets served through beacons can be queried individually or in aggregate via the Beacon Network, a federated search engine (http://www.beacon-network.org) [1]. Thus, the Beacon Project aims to simplify data sharing through a web service (beacon) that provides only allele-presence information. Users can query institutional beacons for information about genomic data available at the institution. For example, an individual could ask the beacon web server about a genome that has a specific nucleotide and the beacon would response either yes or no [4]. By providing only allele-presence information, beacons were assumed safe from attacks that require allele frequencies.

Although the beacon network has set up to share data and protect patient privacy simultaneously, it could potentially leak phenotype and membership information of an individual [4]. There is currently no cap on the number of queries a user can make in the Beacon database. Recently, Shringarpure and Bustamante showed that anonymous patients whose DNA data is shared via beacon network can be re-identified [5]. If an attacker has access to victims DNA, s/he can query different beacons to see whether the victim is in the dataset. They further demonstrated that it is possible to infer whether or not the victim is affected by a certain condition or disease [5]. Therefore, the anonymous beacons are inherently insecure and are open to re-identification attacks. For brevity, we will denote the attack as *Bustamante Attack* through the rest of the paper.

Very recently, some solutions [6, 7] have been proposed based on different policies around the access of the beacon service. However, these solutions will disrupt the quintessential feature of the proposed beacon service: that is to provide faster access to genomic data and to give open access to the research community. Different access controls are highly necessary for human genomic data access where phenotype or sensitive information about the disease is disclosed. However, the beacon service only provides us aggregate results of yes or no leading the researcher to a decision regarding the dataset’s relatedness to his or her research. Therefore, we propose two solutions based on privacy-preserving techniques, which fit well with the beacon service and mitigate the possibility of identifying an individual from the dataset.

In this article, we explain the ‘Bustamante Attack’ [5] on genomic beacon services and propose two privacy preserving solutions. The contributions of this article can be summarized in two folds: *a*) understanding the statistical formulations and soundness of the attack, *b*) analyze lightweight privacy preserving solutions to mitigate the attack. The main contributions of our work are as follows:

- We present the statistical and the mathematical model of the attack in a simplified form. This helps us to analyze different and more realistic parameters on the original attack framework to exploit some weakness and justify our solutions accordingly.
- We show the required steps for any data owner to calculate the risk involved in sharing their genomic data in a beacon service.
- We propose two easy to implement and lightweight privacy preserving solutions which ensure the applicability of the beacon service as well as the privacy of the participants.
- We provide extensive experiments over synthetic data (according to [5]) to show the privacy-utility evaluation of our proposed methods which will help the development of different privacy preserving techniques on such attack model later on.

### Beacon service for genomic data

**Fig. 1:**
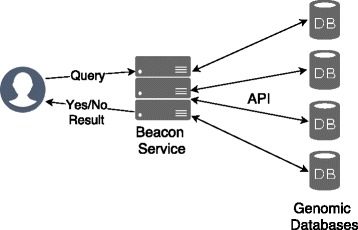
Beacon architecture where researcher and data owners are connected to the central beacon service

A beacon is an online web search engine developed by the Global Alliance for Genomic and Health (GA4GH), which provides a way for genomic data owners and research institutes to easily share genomic data while maintaining patients privacy (Fig. 1). It is a genetic mutation sharing platform that allows any user to query an institution’s databases to determine whether these databases contain a genetic variant of interest while keeping all other sequence data obscured. A query in this search engine is defined by three parameters: chromosome number, position in that chromosome, and target nucleotide (A/T/G/C). A beacon query answer is either true or false, denoting the presence of that nucleotide in that specific position and target chromosome. In other words, it will only answer yes/no for the questions like: Do you have any genomes with an ‘A/T/G/C’ at some position ‘Y’, on specific chromosome ‘Z’. This allows a researcher to target some specific dataset, which is relevant to his or her research. This service also helps a clinician to check whether a mutation found in one of her patients is also present in others without actually having access to their genomes [8].

Beacons are easy-to-implement techniques for several large-scale organizations when it comes to sharing genomic data. It also saves researchers a tremendous amount of time for tracking down useful data for their work as well [9, 10]. Unlike large centralized data repositories, a beacon network is distributed across many databases around the world and is virtually connected through software interfaces allowing continuous authorised access. This federated data ecosystem allows each organization to control their legal data within their jurisdiction [1]. The shared Genomics API in the beacon framework makes it easy to query all at once and ensures that GA4GH team can quickly add new beacons to the network.

### Bustamante attack on beacon service

A recent study done by Shringarpure and Bustamante [5], developed a likelihood-ratio test that uses only allele presence information to predict if the genome of an individual is present or not in the beacon database.This study suggested that beacons are susceptible to re-identification attacks and thus can be subjugated to invade genetic privacy. Since a beacon database includes data with known phenotypes information such as cancer, autism or other diseases, this re-identification also potentially disclose phenotype information about an individual whose genomic data is present in the beacon [11]. Through simulations, they demonstrated that by making just *5000 queries*, it was possible to identify someone and *even their relatives* in a beacon consisting *1000 individuals*. They found that re-identification of an individual is possible even with the sequencing errors and variant-calling differences. They also demonstrated that a beacon constructed with 65 European individuals from the 1000 genome projects, it is possible to detect membership in the beacon with just 250 SNPs [5].

In this section, we briefly introduce the Bustamante attack and analyze its statistical methods. The goal of this attack is to know whether a genomic sequence *g* belongs to a specific database with the help of the beacon service. To answer this question they considered two hypothesis:

1. Null hypothesis *H*_0_: the query individual is not in the beacon service.
2. Alternative hypothesis *H*_1_: the query individual is in the beacon service.

To determine the correct one, the adversary is allowed to query the beacon service with unlimited amount of queries. The adversary queries specific locations where the query individual has alternative allele to see whether the beacon server also contains an individual with the same allele values. Therefore, the responses of the beacon service are a sequence *x*_1_,…,*x*_*n*_ of yes or no. If we consider yes and no with ‘1’ and ‘0’ respectively, the the answer sequence, *R* will be a binary vector. For example, if the query individual is in the database, we will get yes (or 1) in each query. However if there are some genome sequencing error, we might get some wrong answers as well. This error is denoted by *δ* and also considered by the attack [5].

There is also another considerable case where multiple individual have the same allele in the database. This is why the attacker needs to leverage the likelihood ratio of both the assumptions whether the the user is in the dataset or not. For a database of *N* genome, the log of this likelihood ratio can be computed for the response series *R* regarding the hypotheses *H*_*i*_ as follows:

$$\begin{array}{*{20}l} L_{H_{i}}(R)= \sum\limits_{i=1}^{n}x_{i}\log P(x_{i}=1|H_{i})+\\ (1-x_{i})\log P(x_{i}=0|H_{i}) \end{array} $$

where, *n* is the number of queries and *x*_*i*_ is the result from the beacon. *x*_*i*_=1 denotes the query is present in the database which can come either from the target genome or any of the other *N*−1 genomes. *x*_*i*_ is only 0 when the query is not present in any of the *N* genomes.

In article [5], the authors using some simplifying assumptions proved that if the query individual is in the beacon database, *R*=*x*_1_,…,*x*_*n*_ follows a *Binomial* (*n*,1−*D*_*N*_) distribution, otherwise *R* has a *Binomial* (*n*,1−*δ* *D*_*N*−1_) distribution. Therefore, the hypothesis can be rewritten as follows:

1. Null hypothesis *H*_0_: *θ*=*θ*_0_=*n*(1−*D*_*N*_).
2. Alternative hypothesis *H*_1_: *θ*=*θ*_1_=*n*(1−*δ* *D*_*N*−1_).

Therefore, we have:

1 $$ L_{H_{0}}(R)=\sum\limits_{i=1}^{n}x_{i}\log(1-D_{N})+(1-x_{i})\log(D_{N})  $$

and for alternative hypothesis,

2 $$ L_{H_{1}}(R)=\sum\limits_{i=1}^{n}x_{i}\log(1-\delta D_{N-1})+(1-x_{i})\log(\delta D_{N-1})  $$

where *D*_*N*−1_ is the probability that other *N*−1 individuals (all individual except the query individual) have not the specified allele in the determined location.

Basically, the $L_{H_{i}}(R)$ will maximize if the *H*_*i*_ hypothesis is correct. Therefore, we compute $\Lambda =L_{H_{0}}(R)-L_{H_{1}}(R)$ and the *Λ* will declare which hypothesis is true.

The log of the likelihood-ratio statistics can be rewritten from Eqs. 1 and 2 as,

3 $$\begin{array}{*{20}l} \Lambda &= L_{H_{0}}(R)- L_{H_{1}}(R) \\ &=n\log\left(\frac{D_{N}}{\delta D_{N-1}}\right)+ \log\left(\frac{\delta D_{N-1} (1-D_{N})}{D_{N} (1-\delta D_{N-1})}\right) \sum\limits_{i=1}^{n}x_{i} \\ &=nB+C\sum\limits_{i=1}^{n}x_{i} \end{array} $$

In any distribution, a threshold *t* can be fixed where the null hypothesis will be rejected if *Λ*<*t* and accepted otherwise. The attacker need to decide an appropriate threshold for a specific beacon dataset before launching the attack. Suppose a false positive error *α* is given. Regarding this value and the beacon statistical properties, the threshold *t*_*α*_ is determined such that *Pr*(*Λ* < *t*_*α*_|*H*_0_) = *α*. From Eq. 3,

4 $$\begin{array}{@{}rcl@{}} &Pr\left(nB+C\sum_{i=1}^{n}x_{i}<t_{\alpha}|H_{0}\right)<\alpha \\ &Pr\left(\sum_{i=1}^{n}x_{i}>\frac{t_{\alpha}-nB}{C}|H_{0}\right)<\alpha~(C~is~negative) \\ &Pr\left(\sum_{i=1}^{n}x_{i}>t'_{\alpha}|H_{0}\right)<\alpha \end{array} $$

In the attack instead of calculating *Λ* and comparing it to the threshold *t*_*α*_, $\sum _{i=1}^{n}x_{i}$ is computed and compared with $t^{\prime }_{\alpha }$ to make the decision. This threshold $t^{\prime }_{\alpha }$ is used to decide whether the null or the alternative hypothesis is correct. In other words whether the individual is present in the beacon database or not will be dictated by this $t^{\prime }_{\alpha }$. To calculate this, the adversary sums the responses from the beacon *x*_*i*_ and retrieves $\sum x_{i}$. The null hypothesis is rejected simply if $\sum x_{i}>t^{\prime }_{\alpha }$ which leads to a conclusion that the query individual is present in the beacon and the attack is successful.

To calculate the *D*_*N*_, the authors assumed that the adversary has an idea about the distribution of the allele frequencies on those query positions. Specifically, alternate allele frequencies, *f* for all SNPs observed in the population are claimed to be distributed as a *β* distribution according to [5]. Here, *f*∼*β*(*a* ^′^,*b* ^′^), where *a*=*a* ^′^+1 and *b*=*b* ^′^+1, and (*a* ^′^,*b* ^′^) can be precomputed from the genomic dataset in which the beacon service is running. Thus, the adversary needs $\phantom {\dot {i}\!}n\sim N^{a^{\prime }+1}$ queries to make his or her decision whether the target individual is present in the database. The value *D*_*N*_ can be approximated as,

5 $$ D_{N}\approx \frac{\Gamma(a+b)}{\Gamma(b)(2N+a+b)^{a}}  $$

To see the details of deriving and proving the above formula see [5]. We will need this $t^{\prime }_{\alpha }$ and *D*_*N*_ for further analysis in the upcoming section as these parameters dictate the attack.

## Methods

In this section, we provide an analysis of Eqs. 4 and 5 to calculate $t^{\prime }_{\alpha }$ before describing our privacy preserving solutions and experimental results. This analysis allows the beacon service providers and data owners to calculate the risk involved while sharing their beacon data.

### Risk analysis of a beacon service

In this section, we evaluated $t^{\prime }_{\alpha }$ in greater depth for analyzing the risk involved for a specific beacon dataset. Given *N* samples and *n* number of queries, this analysis will help us to determine the number of correct answers that can be returned without identifying the victim.

In other words, as $t^{\prime }_{\alpha }$ directly effects the decision boundary of the correctness of null or alternative hypothesis, its better to theoretically ratify its value on a specific setting. We simulated this on some real life human genomic databases like 1000 Genomes Project, SSMP [12] and GoNL [13] for better understanding.

According to central limit theorem the value $R_{e}=\frac {1}{n}\sum _{i=1}^{n}{x_{i}}$ follows normal distribution $\mathcal {N}\left (1-D_{N}, \frac {D_{N}(1-D_{N})}{n}\right)$.

The threshold $t^{\prime }_{\alpha }$ can then be calculated from Eq. 4 as follows:

$$\begin{array}{@{}rcl@{}} &Pr\left(\sum_{i=1}^{n}x_{i}>t^{\prime}_{\alpha}|H_{0}\right)=\alpha\\ &\Rightarrow Pr\left(\frac{1}{n} \sum_{i=1}^{n}x_{i}>\frac{t'_{\alpha}}{n}|H_{0}\right)=\alpha\\ &\Rightarrow Pr(R_{e}>t^{\prime\prime}_{\alpha}|H_{0})=\alpha \end{array} $$

where $t^{\prime \prime }_{\alpha }=\frac {t^{\prime }_{\alpha }}{n}$. We know that *R*_*e*_ follows the $\mathcal {N}\left (1-D_{N},\frac {D_{N}(1-D_{N})}{n}\right)$ distribution where *θ*_0_=1−*D*_*N*_ and variance $\sigma ^{2}_{0}=\frac {D_{N}(1-D_{N})}{n}$. Therefore, $\frac {Re-\theta _{0}}{{\sigma _{0}}}$ has standard normal distribution, $\mathcal {N}(0,1)$. Suppose we want to have a *α*=0.05 false positive probability then,

6 $$ Pr\left(\frac{Re-\theta_{0}}{{\sigma_{0}}}>\frac{t^{\prime\prime}_{\alpha}-\theta_{0}}{{\sigma_{0}}}\right)=0.05  $$

According to the normal cumulative table and given the fact that $\frac {Re-\theta _{0}}{{\sigma _{0}}}$ follows standard normal distribution, we have,

$$\begin{array}{@{}rcl@{}} &\frac{t^{\prime\prime}_{\alpha}-\theta_{0}}{{\sigma_{0}}}=1.65\Rightarrow t^{\prime\prime}_{\alpha}=1.65\sigma_{0}+\theta_{0}\\ &\Rightarrow t^{\prime}_{\alpha}=n(1.65\sigma_{0}+\theta_{0})=n\left(1.65\frac{\sqrt{D_{N}(1-D_{N})}}{\sqrt{n}}+\theta_{0}\right) \end{array} $$

Table 1 shows the computation of the $t^{\prime }_{\alpha }$ for *α*=0.05 in different beacon databases constructed from real life genomic datasets according to [5].

**Table 1:** Evaluating the threshold $t^{\prime }_{0.05}$ for three different databases with *δ*=10^−3^ mismatch error

Beacon database	Number of records *N*	*a* ^′^	*b* ^′^	*D*_*N*_	$t^{\prime }_{\alpha }$
1k Genomes Phase 1	1092	0.0735	1.0096	0.0005594974767507827	1826
1k Genomes Phase 1 Affymetrix	1074	0.6483	1.2876	1.5352703647724165e-05	99084
GoNL	498	0.1131	0.8574	0.0009412979457329326	1005
SSMP	100	0.1848	0.8500	0.00403048895537907	234
Simulation	2000	0.1178793	1.1188360	0.00022374264418961542	4900

The threshold $t^{\prime }_{\alpha }$ indicates the number of *yes* that an adversary requires to conclude that the query individual is within the dataset. For example in Table 1, the experimental results show that for ‘1k Genomes Phase 1’ dataset with 1092 individuals, the adversary needs 1826 *yes* answers of queries to infer that the victim is present in the dataset. Any quantity less than 1826 yes answers (with mismatch rate *δ*=10^−3^) will conclude that the individual is not present.

**Fig. 2:**
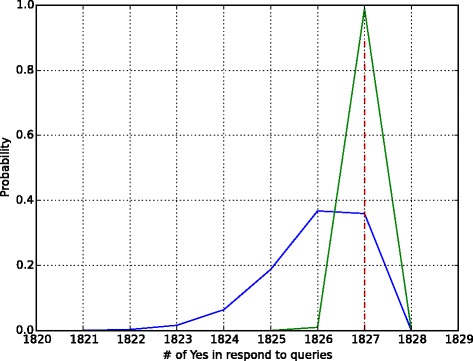
Risk analysis of data in any genomic dataset. The *green* line represents the responds of the beacon service when the query individual being in beacon database while the *black* one represents them not being there. The *red* line denotes the $t^{\prime }_{\alpha }$

The relationship between null and alternative hypothesis along with the threshold are showed in Fig. 2. In Fig. 2, the black and the green lines represent the outputs distributions. If the null hypothesis is true, then the outputs follows black line and the outputs follows the green line if the alternative hypothesis holds. Three other real world datasets were tested and depicted in the Additional file 1.

However, regardless of the risk analysis of a specific dataset with the outlined equations, the beacon service still needs privacy preserving mechanisms. The necessity of such methods are amplified due to the fact that these beacons are designed to support thousands of public queries. Though the simplified equations above can enlighten a data owner about the sensitivity of the underlying data, the data owner still needs some methods, which we will present in the next section, to protect the privacy of the individuals.

### Proposed methods

In this section, we propose two privacy preserving methods which are similar in nature. However the probability of different outputs from these two methods are different. The methods are:

1. Method 1: Eliminating random positions.
2. Method 2: Biased randomized response.

Both of these methods introduce inaccurate results to hide the presence of any individual in a beacon web service. Due to this inaccuracy, these methods destroy some utility of the beacon service as the underlying data will be perturbed. Hence, we need to devise methods that give incorrect answers (false positives or negatives) as less as possible. A false positive is answering *yes* when the query result is *no*. Similarly, a false negative is answering *no* when the query result is *yes*. In “Results” section, we experimentally evaluate these two methods in terms of data privacy and utility.

#### Eliminating random positions

In this method, the data owners apply Algorithm 1 to output their data to the beacon service provider. The output of Algorithm 1 will infuse inaccuracies in the result from the original data with the help of a driving factor *bias*. For example, if data owner has a dataset $\mathcal {D}$ then this method will transform it to a $\mathcal {D^{\prime }}$ where there will be some false positives and negatives with respect to bias, *b*.



In Algorithm 1, for higher bias value, we will get higher accuracy and for lower bias value, we will get lower accuracy. For example, if the bias value is 50, we will obtain answers with a probability of 50% false positives and negatives. We further analyzed different bias value for this algorithm in “Results” section.

#### Biased randomized response

Randomized response [14] was proposed in 1965 by Warner as a statistical tool to remove potential bias and add a probabilistic noise to the answers. For example, data owners transform $\mathcal {D}\rightarrow \mathcal {D^{\prime }}$ with respect to certain probability. In the original method, the person who has been asked a private question flips a coin. If it is tail then s/he answers truthfully. Otherwise, for head, s/he flips the coin again and responds truthfully for tail and provides opposite answer for head.

In a beacon service, we incorporated this method as beacon queries are considered private and their answers are in binary (yes or no) form. For example, a typical query inquires about the presence of a major and minor allele in a specific position of a chromosome. Algorithm 2 can transform the raw data according to the randomized response method and this transformed data can be used further to answer queries.

However, answering queries in this fashion will induce some error and the utility of the beacon services will be at question. Thus, we experimented on a biased randomized response where this 1/2 probability is modified for better utility or true results in Algorithm 2.

In Algorithm 2, we changed the dichotomous behaviour of general randomized response with a control variable named bias. Similar to Algorithm 1, a higher bias will give more accurate result and will provide less privacy on the data. We showed the analysis for different bias values in “Results” section.

However, there is a similarity between both the algorithms. Algorithm 1 returns true answer with probability *b* given *b*∈[0,1], while Algorithm 2 returns true answer with probability 1−(1−*b*)^2^. Therefore, Algorithm 2 with bias *b*_2_ will be same as Algorithm 1 having bias $b_{1} = 2b_{2}-b_{2}^{2}$ where *b*_1_,*b*_2_∈[0,1].



##### **Theorem 1**

1 The response from Algorithm *2* is $|ln\left (\frac {1}{(1-b)^{2}}-1\right)|$ differentially private.

##### *Proof*

Lets fix a respondent and a randomized device (i.e., coin flip) with bias *b* and range [ 0,1]. For a ‘Yes’ answer from this respondent, we get

$$\begin{array}{*{20}l} P(Response=Yes | Truth = Yes) = b+(1-b)b\\ P(Response=Yes | Truth = No) = (1-b)^{2} \end{array} $$

Thus for ‘Yes’ answer we have,

$$\begin{array}{*{20}l} \frac{P(Response=Yes | Truth = Yes)}{P(Response=Yes | Truth = No)} =\frac{b+(1-b)b}{(1-b)^{2}} \\ =\frac{1-(1-b)^{2}}{(1-b)^{2}} = \frac{1}{(1-b)^{2}}-1 \end{array} $$

Similarly for a ‘No’ answer we have,

$$\begin{array}{*{20}l} \frac{P(Response=No | Truth = No)}{P(Response=No | Truth = Yes)}&=\frac{1}{(1-b)^{2}}-1 \end{array} $$

Since, both the probabilities are bounded by $\nobreak {\frac {1}{(1-b)^{2}}-1}$, Algorithm 2 satisfies $|ln\left (\frac {1}{(1-b)^{2}}-1\right)|$ differentially privacy. □

For example, if the randomized device is a regular coin then we have bias $b=\frac {1}{2}$ in range [ 0,1]. Thus the mechanism would be $|ln\left (\frac {1}{(1-\frac {1}{2})^{2}}-1\right)|=|ln(3)|$ differentially private [15].

## Results

In this section, we evaluated both the methods according to the proposed attack [5]. We used similar experimental setup according to the original paper to directly benchmark our solution to the attack scenario. We also changed some of their population size and other parameters in order to do further analysis.

### Original results

The original simulation [5] were experimented on a sample of 1000 individuals containing 500,000 SNPs which we doubled to 1,000,000 SNPs. Alternate allele frequencies of these SNPs were sampled from binomial distribution for a standard neutral model under the assumption of a population size of 20,000 individuals. Then the query beacon was constructed having 1000 individuals (from 20,000). We also considered higher beacon size with 1200,1500,2000 individuals. Then the log likelihood ratio tests (LRT) to confirm the hypothesis were done assuming,

- 400 individuals from the beacon.
- 400 individuals not from the beacon.

The comparison between both setups are also shown in Table 2.

**Table 2:** Parameter consideration in our experiment and the original paper [5] (1k=1000)

Parameter name	Original paper [5]	Our setup
Population size	10k	20k
SNPs considered	500k	1,000k
Beacon size	1k	1k, 1.2k, 1.5k, 2k

**Fig. 3:**
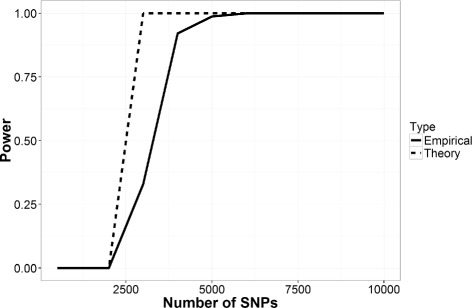
Power (LRT) of re-identification attacks of individuals on beacons constructed with 1000 individuals on our experimental setting without any privacy preserving mechanism

The outcome of the attack in our setting is depicted in Fig. 3 where the power of the log-likelihood ratio tests (LRT) are on *Y* axis while the *X* axis shows the number of SNPs queried by any adversary. The figure demonstrates that the proposed attack has more than 95% power to detect whether an individual is present in the beacon of 1000 individuals with just 5000 SNP queries. This result also supports the claim of the original paper [5].

**Fig. 4:**
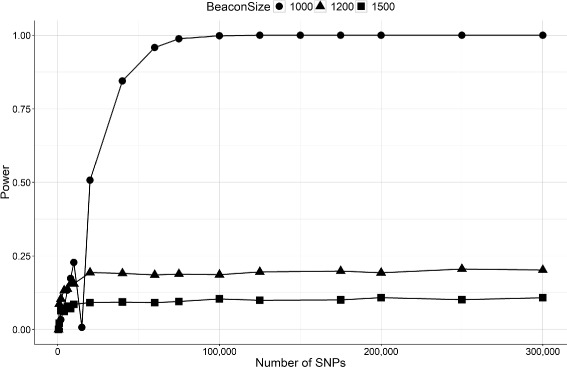
Power (LRT) of re-identification attacks on beacons constructed with different number of individuals. We show the results of the attack for 3 different beacon database size (1000,1200,1500)

As Eq. 5 shows the dependency between the LRT outputs and the beacon size (number of individuals, *N*), we further analyzed the attack for a different number of *N*. We show the power of the attack for different beacon size *N*={1000,1500,2000} in Fig. 4.

In the Additional file 1, we include the analysis for different genome sequencing error rate and re-identifying the relatives of those 400 individuals. For example, the relatedness (*ϕ*) can be defined as twins, parent-offspring, siblings, cousins etc., where *ϕ*={1,0.5,0.25,0.125}. As twins share the same genomic sequence, the LRT tests should be similar and conclusive after 5000 queries.

### Our results

According to the test framework, we evaluated our proposed methods. We employed our privacy preserving mechanisms to perturb the original answer and then evaluated the performance of our techniques.

**Fig. 5:**
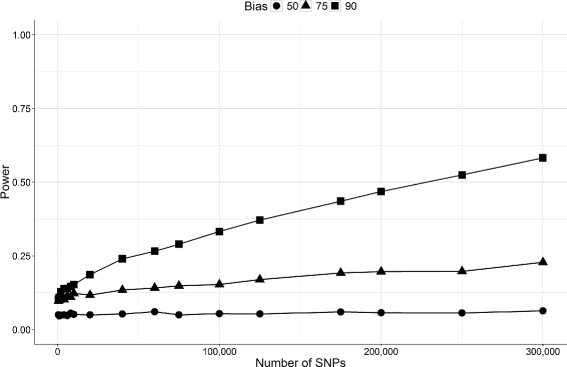
Effect of method 1 on the power of the attack on beacon database considering different bias

**Fig. 6:**
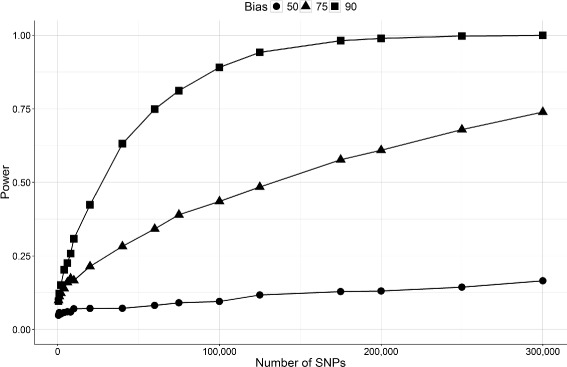
Effect of method 2 on the power of the attack on beacon database considering different bias

Figures 5 and 6, show the results of Algorithm 1 and 2. As expected from the privacy-utility relation, we see that more accurate answer results in less privacy as the LRT powers keep rising for bias 90 (90% accuracy) after 300,000 queries. That is even with only 10% errors, the adversary needs more than 300,000 queries to determine the presence of an individual in the beacon database.

### Accuracy analysis

As mentioned previously, there is a need for a method which will induce errors to provide the privacy of the individuals’ present in a beacon service. Both of our methods add random errors to the beacon database where these errors can be defined as false positives and negatives. In this context, *false positives* are those where the beacon service answered yes regardless of the fact that there was no existence of that data. *False negatives* are those where the beacon answered false to a true answer. Accuracy is defined as,

$$\begin{array}{*{20}l} accuracy = \frac{N_{TP}+N_{TN}}{N_{TP}+N_{TN}+N_{FP}+N_{FN}} \end{array} $$

**Fig. 7:**
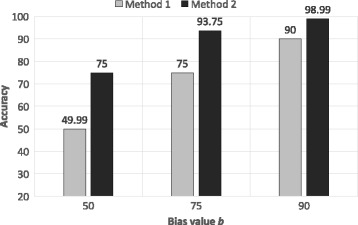
Accuracy of both methods with different bias (50, 75, 90) consideration

Figure 7 shows the calculation of the accuracy for both of our methods. It is clear from the figure that both methods with a higher bias provide more accurate result. This allows the corresponding data owner to decide the amount of utility they want to provide with respect to the privacy of the individuals.

**Fig. 8:**
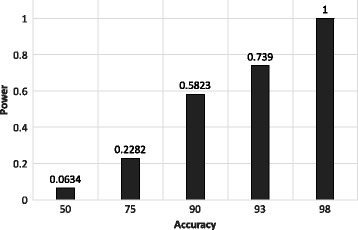
Privacy-Utility curve for different accuracy on X-axis and their corresponding LRT powers for 300,000 queries

We also show multiple levels of privacy achieved for different accuracy of the beacon data. Figure 8 shows the different LRT powers for different accuracy of the data in 300,000 queries. As higher LRT powers defines better assumptions from the adversary, we can model it as the privacy loss where utility can be defined as the accuracy aforementioned. It is noteworthy that, higher utility results in higher privacy loss as we can see with 98% accuracy we have LRT power as 1 where 75% accuracy has 0.22.

## Discussion

In this section, we discuss few issues regarding the original attack and the applicability of our solutions.

### Different bias on tiered access control

One clear indication from GA4GH and the research community on this privacy issue of genomic beacon service is implementing an access control over this sensitive information [1, 7]. Multiple layers of access control have been proposed where a different level of users will have different privileges over the beacon service. This kind of hierarchy in accessing a service is often named as ‘tiered access control’. The applicability of this model in beacon service is already proposed in a recent study [6].

Our solution methods fit the tiered access control as we have different levels of privacy guarantee for different bias value. Higher bias leading to higher accuracy might be granted to a more trusted user where a public user might only get the lowest utility with high privacy over the beacon data. This will ensure the utility that the beacon promises while not revealing the presence of an individual.

### Statistical inference attack

There are two different ways we can incorporate our methods on a beacon service. First, by using them while answering queries in real time and secondly, using them to preprocess the database beforehand to answer queries. In our analysis, we use the algorithms to preprocess the database due to the statistical inference attack. If we use the algorithms in real time, then an adversary might average the outputs of a specific query and obtain the original output.

### Different allele frequency assumptions

The original attack scenario assumes that the allele frequency of the dataset follows a beta distribution [5]. However, in real life, an adversary can find the specific allele frequency of any position from a public database. This enables the adversary to launch more powerful attacks against the beacon service. It is noteworthy that iDASH 2016 competition [16] presented the problem under this formulation [17, 18].

However, in this paper we also assume that the adversary has limited background knowledge and s/he does not have access to specific frequencies of each position. More rigorous privacy guarantee like *differential privacy* [15] can be provided against a stronger adversary.

## Related work

Genomic privacy has recently gained significant concern among the general public and research community. De-identification is a common practice in research and clinical practice to protect genomic privacy and confidentiality of the participants. Normally, privacy is achieved by anonymizing a person’s identity while sharing genomic related data. Since the de-identified genomic data are typically published with additional metadata and anonymized quasi-identifiers information, these pieces of information can be used to re-identify an unknown genome and thus disclosing the identity of the participant. Significant research has been done so far in this area. Below are some of the recent works related to re-identification attacks in genomic and health-related data.

In the recent study, Sweeney et al. [19] showed that participants in the Personal Genome Project (PGP) can be easily identified based on their demographics without even using any genomic information. They also stressed that 84 to 97% of the participants are correctly identified by linking the demographics to publicly available records such voter list and the name hidden in the attached documents.

Gymrek et al. [20], showed that a person’s identity can be exposed via surname inference by profiling short tandem repeat on the Y-chromosome and querying recreational genomic genealogy databases. In their study, they showed that by scanning two largest Y-chromosome genealogical websites, 10–14% US white male individuals are subject to surname inference attack. Moreover, when the attacker gains access to that target DNA sample, they can simply search available genomic databases with sensitive attributes (e.g., drug abuse). Hence, the person’s identity with attributes can be easily found.

In recent study [21], Gitschier showed that a surname of an individual participating in HapMap database can be inferred by the combination of information from genealogical registries and a haplotype analysis of the Y-chromosome collected for the HapMap Project. In [22], the authors presented an attack that involves the association of DNA sequences to personal names, through diagnosis codes.

Zhou et al. [23] studied the privacy risks of releasing aggregate genomic data and showed that individuals participating in such research study can be easily identified and for some cases, their DNA sequences can be fully recovered. They have proposed a risk-scale system to classify aggregate data and a guide for their release.

Homer et al. [24] proved it is possible to detect the presence of an individual in a complex genomic DNA mixture even when the mixture contains only trace quantities of his or her DNA.They showed that an individual participating publicly released Genome Wide Association Study (GWAS) can be easily identified by his/her known genotypes and analysing the allele frequencies of a large number of SNPs.

Wang et al. [25] showed a higher risk that individuals can actually be identified from a relatively small set of statistics such as those routinely published in GWAS papers. Their first attack is the extension of homer’s attack and showed that the presence of an individual in the case group can be determined based upon the pairwise correlation among as few as a couple of hundred SNPs. The second attack can lead to a complete disclosure of hundreds of the participants’ SNPs, by analyzing the information derived from the published statistics.

In another study, Malin and Sweeney in [26] introduced re-Identification of Data in Trails (REIDIT) algorithms which link individuals genomic data to the publicly available records. They showed that it is possible to identify a person by looking at the unique features in patient-location visit in a distributed healthcare environment.

Other than these, there are multiple surveys available which summarizes and demonstrates some other attacks [27, 28].

## Conclusion

Bustamante attack on beacon service presents a privacy problem of sharing genomic data publicly and demonstrates the need for further research to achieve genomic data privacy. In this paper, we analyzed Bustamante attack and provided a method to calculate the risk involved in sharing the genomic data. We proposed two simple privacy preserving solutions: eliminating random positions and biased randomized response. Our lightweight privacy preserving solutions ensure a good trade-off between data privacy and utility. Experimental results demonstrate that given higher bias, both the methods are able to provide high data utility.

## Additional file

Additional file 1 Contains further analysis on the attack and the solution mechanisms. (PDF 334 kb)
